# A Coarse-Grained SPICA Makeover for Solvated and Bare Sodium and Chloride Ions

**DOI:** 10.1021/acs.jctc.4c00529

**Published:** 2024-08-19

**Authors:** Janak Prabhu, Matteo Frigerio, Emanuele Petretto, Pablo Campomanes, Stefan Salentinig, Stefano Vanni

**Affiliations:** † Department of Biology, University of Fribourg, 1700 Fribourg, Switzerland; ‡ Department of Chemistry, University of Fribourg, 1700 Fribourg, Switzerland; § National Center of Competence in Research Bio-inspired Materials, University of Fribourg, 1700 Fribourg, Switzerland

## Abstract

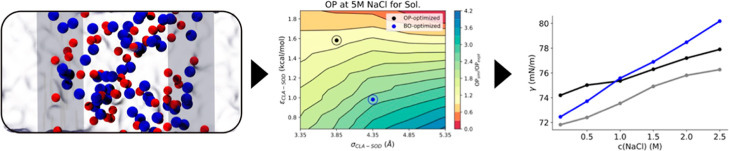

Aqueous ionic solutions are pivotal in various scientific domains due to their natural prevalence and vital roles in biological and chemical processes. Molecular dynamics has emerged as an effective methodology for studying the dynamic behavior of these systems. While all-atomistic models have made significant strides in accurately representing and simulating these ions, the challenge persists in achieving precise models for coarse-grained (CG) simulations. Our study introduces two optimized models for sodium and chloride ions within the nonpolarizable surface property fitting coarse-grained force field (SPICA-FF) framework. The two models represent solvated ions, such as the original FF model, and unsolvated or bare ions. The nonbonded Lennard-Jones interactions were reparameterized to faithfully reproduce bulk properties, including density and surface tension, in sodium chloride solutions at varying concentrations. Notably, these optimized models replicate experimental surface tensions at high ionic strengths, a property not well-captured by the ions of the original model in the SPICA-FF. The optimized unsolvated model also proved successful in reproducing experimental osmotic pressure. Additionally, the newly reparameterized ion models capture hydrophobic interactions within sodium chloride solutions and show qualitative agreement when modeling structural changes in phospholipid bilayers, aligning with experimental observations. For aqueous solutions, these optimized models promise a more precise representation of the ion behavior.

## 1 Introduction

Aqueous ionic solutions are pivotal in studying batteries, chemical processes, and biological systems.^1−3^ Notably, at a cellular scale, these solutions with dissolved ions influence cellular functions by interacting with proteins and phospholipid membranes, for example, impacting membrane fusion^4,5^ and regulating ion channels.^6,7^ Ions also play a pivotal role in the hydrophobic effect,^8,9^ modulating the aggregation of nonpolar molecules in water, with direct implications for fundamental biological processes like protein folding and biomolecular self-assembly. Notably, the positioning of sodium and chloride ions at the upper end of the Hoffmeister series enhances the hydrophobic nature of nonpolar molecules in sodium chloride solution.^10^ Additionally, the balance of ionic concentrations in the intra- and extracellular environments, as well as its osmotic regulation by semipermeable barriers such as cellular membranes, is essential for pH regulation that aids multiple metabolic processes.^11,12^

Generally, these phenomena are dynamically driven processes that take place at scales that are not easily accessible experimentally. Therefore, simulation techniques such as molecular dynamics (MD) are often employed to derive a mechanistic explanation. In this regard, force fields (FFs) play a crucial role in MD simulations, and selecting an appropriate FF influences the accuracy of the simulation results. To this end, multiple FFs have been developed for all-atom (AA)^13,14^ and coarse-grained (CG)^15,16^ simulations to describe aqueous solutions with dissolved ions. AA-FFs have proven effective in representing ionic solutions, with numerous studies refining and improving their reliability.^17−20^ On the other hand, CG simulations offer computational efficiency by reducing the degrees of freedom.^21^ However, this leads to a loss of accuracy since CG-FFs simplify the interactions between molecules by condensing high-resolution details into a few parameters.^22^ Hence, careful optimization of the parameters describing nonbonded Coulombic and Lennard-Jones (LJ) interactions and their validation against either higher-resolution AA simulations or experimental data is essential.

Typically, CG-FFs describe multiple water molecules as a single bead. These models, which are referred to as nonpolarizable water models, have found widespread use in various studies due to their computational efficiency.^15,23^ However, the situation becomes complicated when it comes to modeling ions. This arises from the significant role that charges play in the behavior of ions. For instance, simple LJ interactions alone fall short of capturing the behavior of ions, as the net interaction between ions can be overwhelmingly repulsive if they bear the same charge or attractive for opposite charges. To this end, it becomes imperative to treat Coulombic interactions as distinct entities in CG-FFs when modeling ions to allow for a thorough representation of their behavior within the simulated environment.^15,16^

Among existing chemical-specific CG-FFs, the nonpolarizable surface property fitting coarse-grained (SPICA) FF framework^16,23,24^ is promising for describing biomolecules as it accurately captures thermodynamic properties, such as density and surface tension. This model has shown applicability in reproducing molecular phase separation^24,25^ and complex biomolecular phenomena on large scales, particularly those influenced by surface properties.^26−28^ An example in this context is the high accuracy obtained while simulating the surface pressures in Langmuir monolayers.^24,29^

However, in the case of sodium and chloride ions, certain shortcomings are noticeable. While the FF effectively predicts density and follows the expected trend with increasing salt concentration, it fails to reproduce the correct surface tension trend accurately.^16^ Furthermore, the original work on the SPICA-FF ion model tested the parameters by comparing the CG simulation results with those of AA simulations. However, the original work did not evaluate its performance against experiments such as the osmotic pressure exhibited by sodium chloride solution on a semipermeable membrane.^30,31^ Since osmosis plays a vital role in maintaining homeostasis of ion concentrations in the intra- and extracellular environments in *in vivo* systems,^32^ it is critical to reproduce the osmotic pressure of ionic solutions at various concentrations. Additionally, it is known that the structural properties of phospholipid bilayers vary in the presence of monovalent ions.^33^ Therefore, ample space is available for further improvement in the description of ions within the SPICA-FF framework.

In this work, two newly parameterized models for ions, based on a systematic top-down parameterization strategy, are compared against the original SPICA-FF. Our strategy involved fine-tuning the LJ potential’s nonbonded parameters (ε and σ) using a Bayesian optimization (BO) procedure to reproduce the experimental density and liquid–vapor surface tension of sodium chloride solutions, while keeping the Coulombic interaction parameters unchanged. Contrary to the protocol used to obtain the current SPICA-FF ionic parameters, which involved the reproduction of density and surface tension in a narrow range of ionic concentrations, the parameters here developed were obtained by fitting various experimental properties in a wider range of concentrations. The first model implements the same solvated ion mapping scheme as that of the original FF, where each ion is grouped with water molecules to form an ion bead. The second model introduces bare ions (without grouped water molecules) into the FF. To avoid a large-scale optimization of the parameters currently present in the FF, we maintained the same screened charged approach employed to parameterize the original FF and left unchanged the Coulombic interaction parameters. Instead, the extensive parameter space provided by the LJ potential was explored to fit the results against two imposed conditions. First, condition 1, where the macroscopic properties, such as density and surface tension with increasing concentration, are reproduced. Second, condition 2, where the osmotic pressure with the increase in concentration is reproduced. Finally, the ability of these newly optimized models to reproduce other experimental results was investigated, highlighting that the newly optimized parameters can accurately replicate interfacial tension on aqueous solution–alkane/oil biphasic systems,^34,35^ and of bilayers in the presence of sodium and chloride ions.^33^

## 2 Methods

### 2.1 Experimental Details

Surface and interfacial tension measurements were conducted at 20 ± 1 °C using a Krüss DSA30 Drop Shape Analyzer (Krüss Scientific, Hamburg, Germany). Sodium chloride (Acros Organics, Geel, Belgium, purity = 99.5%) aqueous solutions (HPLC gradient grade water, Fisher Scientific, Loughborough, United Kingdom) were prepared by dilution from a 5 M stock solution. The aqueous droplets were hanging at the tip of a NE94 steel needle with a 1.8 mm diameter (Krüss Scientific, Hamburg, Germany). A J-shaped needle is required when a less dense liquid than the surrounding phase is placed in the syringe as the drop is upside down. Decane (Thermo Scientific, Kandel, Germany, purity 99+%) and triolein (Sigma-Aldrich, St. Louis, Missouri, purity 99%) were injected through a 2 mm diameter J-shaped needle (Krüss Scientific, Hamburg, Germany) in a 20 mm light path quartz cell (Hellma Materials, Jena, Germany) filled with the aqueous solutions. Both needles were mounted on a 500 μL SY20 microsyringe (ILS, Stützerbach, Germany). The droplets were left equilibrating for 10 min before image acquisition. The image of the droplet was evaluated using the ADVANCE software (Krüss Scientific, Hamburg, Germany), and the drop shape curvature was fitted according to the Young–Laplace equation
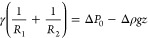
1where γ is the surface or interfacial tension, *R*_1_ and *R*_2_ are the curvature radii of the hanging droplet, Δ*P*_0_ is the Laplace pressure at the interface, Δ*ρ* is the density difference between the air or decane or triolein and the NaCl aqueous solutions, *g* is the standard acceleration of gravity, and *z* is the gap between the measuring point and the needle opening.

Surface and interfacial tension measurements were performed in triplicate, and the mean values with their corresponding standard deviations are presented in Table S5. The densities for the bulk phase were determined separately (Table S6) using a DSA 5000 density meter (Anton Paar, Graz, Austria).

### 2.2 CG Model

The SPICA-FF implements the following functional forms to describe bonded interaction terms
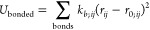

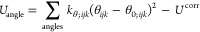

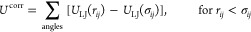
2

where *k*_*b*;*ij*_ and *k*_θ;*ijk*_ are the bond and angular force constants, whereas *r*_0;*ij*_ and θ_0;*ijk*_ are the distance and angle configurations, corresponding to the minimum energy. The *U*^corr^ value based on the LJ potential is added to avoid angle collapses. Nonbonded LJ and Coulombic interaction terms are described as
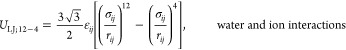

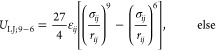

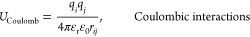
3

### 2.3 Simulation Details

All CG-MD simulations in this study were performed with the Large-scale Atomic/Molecular Massively Parallel Simulator software (LAMMPS).^36^ To ensure temperature control, the Nosé–Hoover thermostat^37,38^ was employed. To maintain pressure during constant pressure simulations, the Nosé–Hoover barostat^37,38^ was utilized. For all simulations, the integration time step size was set to 10 fs as the original FF,^24^ ensuring accurate time evolution of the dynamics. A cutoff distance of 15 Å was applied for the nonbonded LJ interactions. The particle mesh Ewald (PME) algorithm implemented in LAMMPS accounted for long-range electrostatic interactions in SPICA-FF, where a real space cutoff distance of 15 Å was applied. Periodic boundary conditions were implemented in all directions for all of the simulations. All snapshots in this study were rendered with the virtual MD software (VMD).^39^

#### 2.3.1 Bulk Property Simulations

A simulation box was constructed employing the PACKMOL software^40^ to investigate the bulk properties by incorporating water molecules and ions at different concentrations. The number of particles inserted in the initial box ranged from 1000 to 1200 CG beads depending on the ion concentration. The system was equilibrated for 2 ns in the *NPT* ensemble following an initial minimization step. Subsequently, a production run in the *NPT* ensemble was conducted for 20 ns to evaluate the density. All bulk property simulations were conducted at a temperature of 293 K.

Canonical ensemble (*NVT*) simulations were conducted to determine the system’s surface tension. The final snapshot of the density simulation was elongated in the *z* direction to triple its initial length. Following an initial equilibration period of 2 ns in the *NVT* ensemble, a production MD run of 20 ns was performed in the *NVT* ensemble to capture the necessary data for surface tension calculations. The Kirkwood–Irving method^41^ was employed to calculate the surface tension (γ), as follows
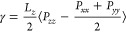
4

where *L*_*z*_ is the length of the box in the *z* direction and *P*_*ii*_ is the pressure tensor diagonal value in the *i* direction. The last 5 ns of the trajectory was used to collect statistics and compute both density and surface tension calculations. Convergence of these profiles over 100 ns was confirmed before finalizing the optimized parameters. One can note that this window suffices for carrying out the simulations to fit the parameters.

To calculate the interfacial tension of the aqueous ionic solutions and alkanes, a simulation box of 1000 decane or 400 triolein molecules and 5000 water + ions was set up as a biphasic system in the *z* direction. The *x* and *y* directions were coupled and fixed. However, the *z* direction was allowed to fluctuate to adjust the normal pressure (NP_N_AT ensemble). Equation 4 was employed to calculate the interfacial tension, while taking the mean of the *z* length of the box in this case.

As a convention, the tension values of the air/liquid and liquid/liquid interfaces are termed as surface tension (γ) and interfacial tension (γ_it_), respectively.

#### 2.3.2 Osmotic Pressure Simulations

**Figure 1:**
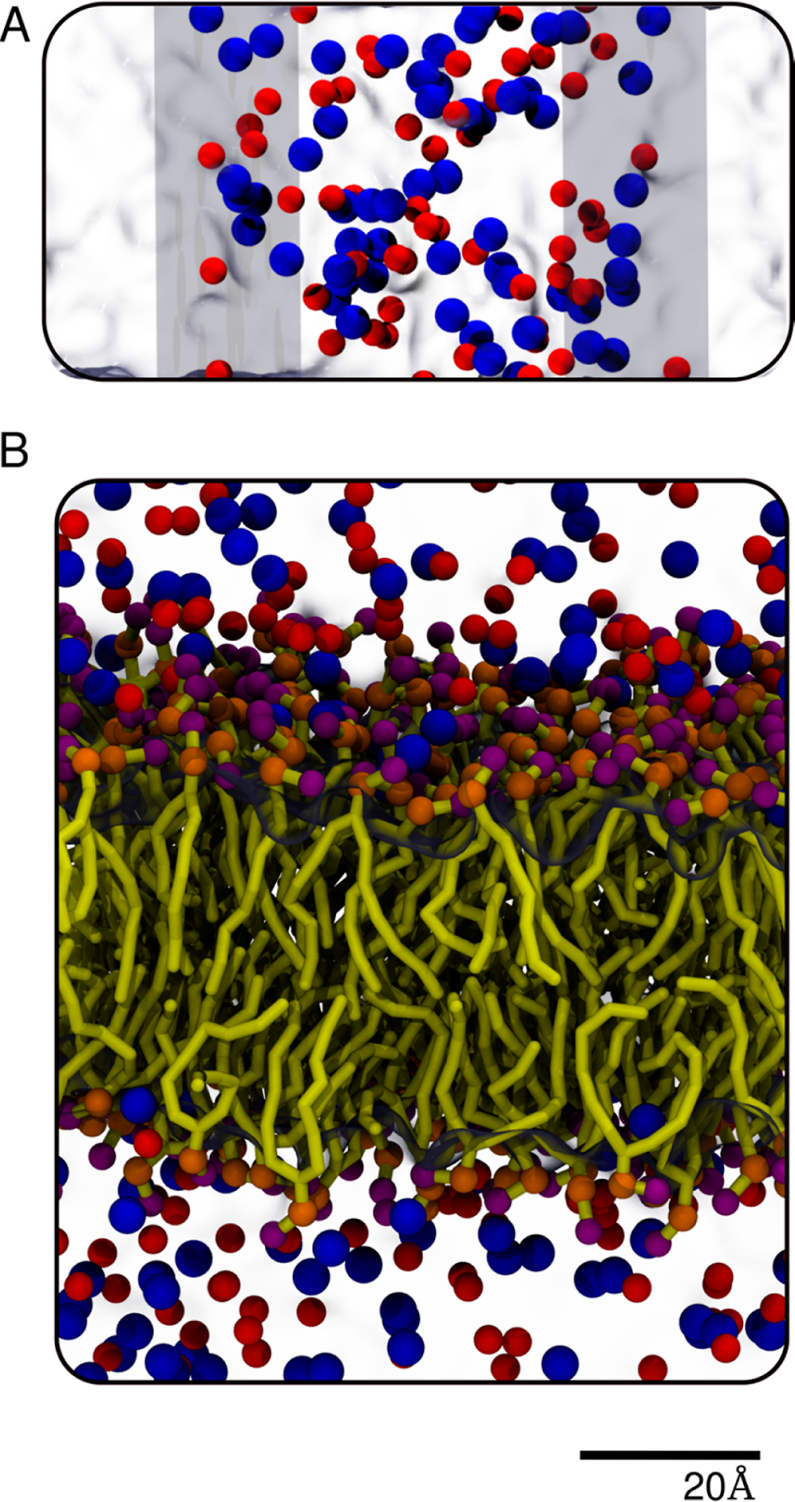
(A) Simulation setup to carry out osmotic pressure (OP) simulations. The blue beads are the sodium ions, and the red beads are the chloride ions. The virtual walls acting as semipermeable membranes are illustrated as ice blue sheets. (B) Representative snapshot, displaying the ion interactions with the POPC bilayer setup (yellow). The positively charged choline group is shown in purple and the negatively charged phosphate group in orange.

Following the previously developed strategy,^20^ an *NPT* bulk simulation with ions was carried out, forming the concentrated phase. Once the density was found to be constant within fluctuations, a water box, without any ions, was added on both sides in the *x* direction to ensure a pure bulk water phase after the equilibration. The concentrated and pure phases were then separated by a virtual wall created by the LAMMPS *fix indent* command. The virtual wall was ensured to act as a semipermeable osmotic membrane by adding flat bottom restraints on the ions if they crossed from the concentrated phase to the clean phase. The water molecules were allowed to flow freely. A representative configuration showing the interface of such a system is provided in Figure 1A. All simulations were conducted at 298 K.

#### 2.3.3 Bilayer Simulations

The pure 1-palmitoyl-2-oleoyl-*sn*-glycero-3-phosphocholine (POPC) and mixed POPC:1-palmitoyl-2-oleoyl-*sn*-glycero-3-phosphoserine (POPS) phospholipid bilayer simulations were simulated in the *NPT* ensemble. The simulation box was generated with PACKMOL. The pressure was handled semi-isotropically, where the *xy* directions (along the bilayer plane) were coupled. The *z* direction (normal to the bilayer plane) was handled separately. These simulations were carried out to calculate the area per lipid (APL) and transverse lipid headgroup–headgroup distance (*D*_HH_; only in the case of POPC) in the presence of ions, as shown in Figure 1B. The APL was calculated as
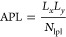
5

where *L*_*x*_ corresponds to the length average in the *x* direction, *L*_*y*_ represents the length average in the *y* direction, and *N*_lpl_ is the number of lipids per leaflet. For POPC bilayer simulations, *N*_lpl_ = 128 and for POPC:POPS bilayer simulations, *N*_lpl_ = 130. *D*_HH_ was calculated as the average transverse distance from headgroup to headgroup. The headgroup was set as the center of mass (COM) of the phosphate and choline CG beads of the POPC phospholipid. All simulations were conducted at 298 K.

The AA bilayer systems were generated from the CHARMM-GUI^42^ package, with equivalent lipid compositions corresponding to the CG systems. The simulations were carried out with the GROMACS 2019.6 software package.^43^ The Parrinello–Rahman barostat^44,45^ was implemented to maintain pressure 1 bar semi-isotropically and the Nosé–Hoover thermostat^38^ was used to maintain temperature 298 K during the production run. The time step was set to 2 fs. The cutoff distance was set to 12 Å with a force switch at 10 Å. Finally, the PME was used to account for the long-range electrostatics.

### 2.4 Osmotic Pressure Method

The methodology of Luo and Roux^20^ was used to calculate the osmotic pressure. First, the osmotic force, denoted as ⟨*F*_wall_⟩, exerted by the ions on the virtual wall was determined by a harmonic equation
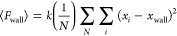
6

Here, *x*_*i*_ represents the position of the *i*th ion when |*x*_*i*_| > |*x*_wall_|, indicating that the ion has crossed the virtual wall. *N* corresponds to the number of frames stored in the trajectory data, and *k* represents the force constant applied on the wall. In this study, we set *k* = 20 kcal/mol/Å^3^. The osmotic pressure (Π_osc_) was then given as
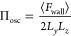
7

where *L*_*y*_ corresponds to the length in the *y* direction and *L*_*z*_ represents the length in the *z* direction.

### 2.5 Scaling of Interactions

To perform simulations with the optimized ionic parameters in the presence of alkane-like moieties, such as in POPC bilayers, the interactions between each bead of these systems and the ionic models were not reoptimized. Instead, we adopted a minimalist approach to facilitate the incorporation of these parameters into the original FF.

In the case of solvated ions, the interactions between the ions and the beads of the phospholipids were left unchanged. These parameters were the same as those in the original FF. For unsolvated or bare ions, instead of explicitly optimizing the interactions, a scaling procedure was employed. Precisely, the LJ interaction parameters were scaled using the following approach
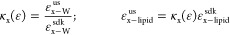

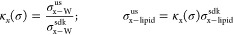
8

Here, *x* represents the sodium or chloride bead and κ is the scaling factor for the LJ interaction parameters ϵ and σ. The values of the scaling factor are provided in the Supporting Information. A similar procedure has been utilized in previous studies for CG-FFs.^46,47^ This scaling procedure allowed for adjustment of the LJ interactions without explicitly optimizing them.

### 2.6 Order Parameters of Phospholipids

To calculate the order parameters, a procedure compatible with the CG-FFs was implemented.^15,24^ The order parameter is calculated as follows
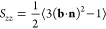
9where **b** is the unit bond vector connecting the two adjacent CG beads in the acyl chain and **n** is the unit bond vector along the bilayer normal. To compare the results from AA simulations, the trajectory of the AA simulations with the CHARMM 36 FF^48^ was converted to the CG trajectory, and the order parameters were calculated with eq 9.

## 3 Results and Discussion

### 3.1 Model Fitting—Density and Surface Tension (Condition 1)

As a first step, the simulation data for density and surface tension were fit to target the experimental data (measured in this work; Tables S5 and S6) across varying concentrations. To do so, the LJ potential’s nonbonded parameters (ε and σ) were tuned by implementing a BO procedure,^49,50^ while Coulombic interaction parameters were left unchanged. These parameters, obtained after carrying out the BO procedure (Tables S1–S4), will be termed as “BO-optimized”. We retained the water bead self-interactions identical to those in the original FF^23^ while reoptimizing all other interactions pertaining to the sodium chloride solution.

**Figure 2:**
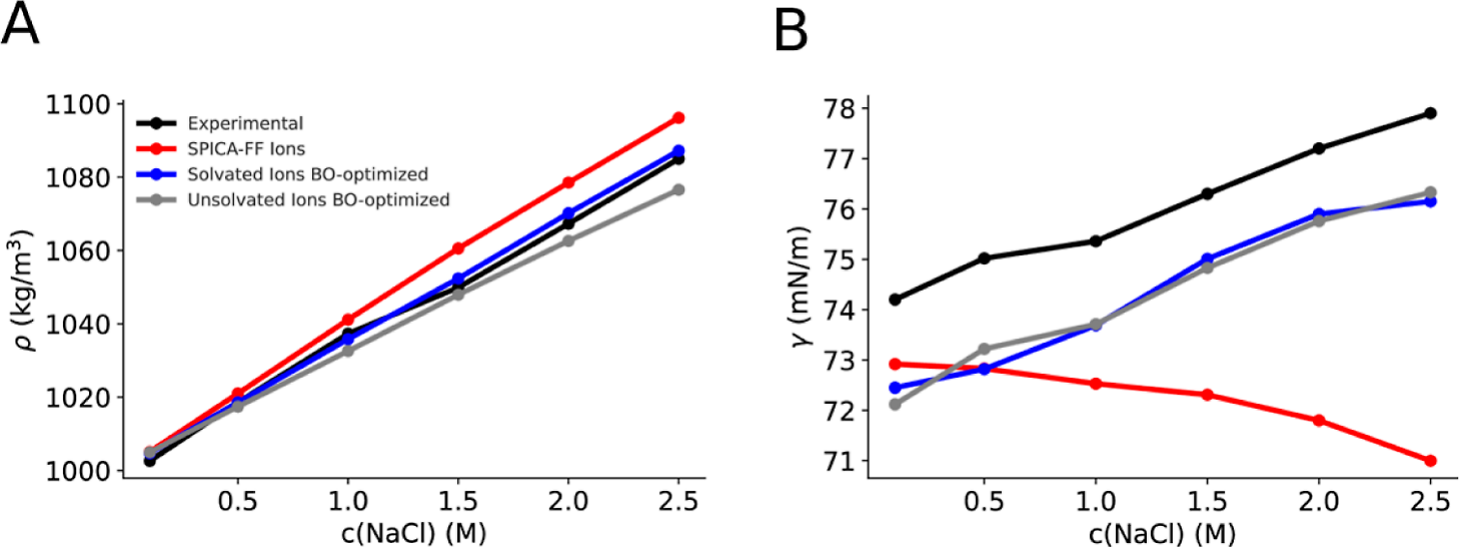
(A) Experimental and simulated density at various concentrations for the ionic models, i.e., the SPICA-FF and the one optimized in the current work. Simulation error ∼1 kg/m^3^. (B) Experimental and simulated surface tension at various concentrations for the ionic models, i.e., the SPICA-FF and the one optimized in the current work. Simulation error ∼0.7 to 1 mN/m.

Figure 2A shows the performance of the different ionic models, including the original SPICA-FF model for ions, in simulating the density evolution of sodium chloride in water. These models capture the density increase and slope with respect to the NaCl concentration in water, aligning well with experimental data. However, Figure 2B highlights a notable disparity in the reproduction of the air/liquid surface tension by the original SPICA-FF ion model, particularly at high sodium chloride concentrations, where it significantly deviates from experimental trends. Encouragingly, the optimized models for solvated and unsolvated sodium and chloride ions exhibit a significant improvement, achieving surface tension reproduction within a 5% margin of error while replicating the observed trend. Furthermore, these results imply that the two presently developed models for ions show a definitive improvement and are more in line with the SPICA-FF standard developmental procedure of reproducing the experimental density and surface tension.

### 3.2 Model Fitting—Osmotic Pressure (Condition 2)

**Figure 3:**
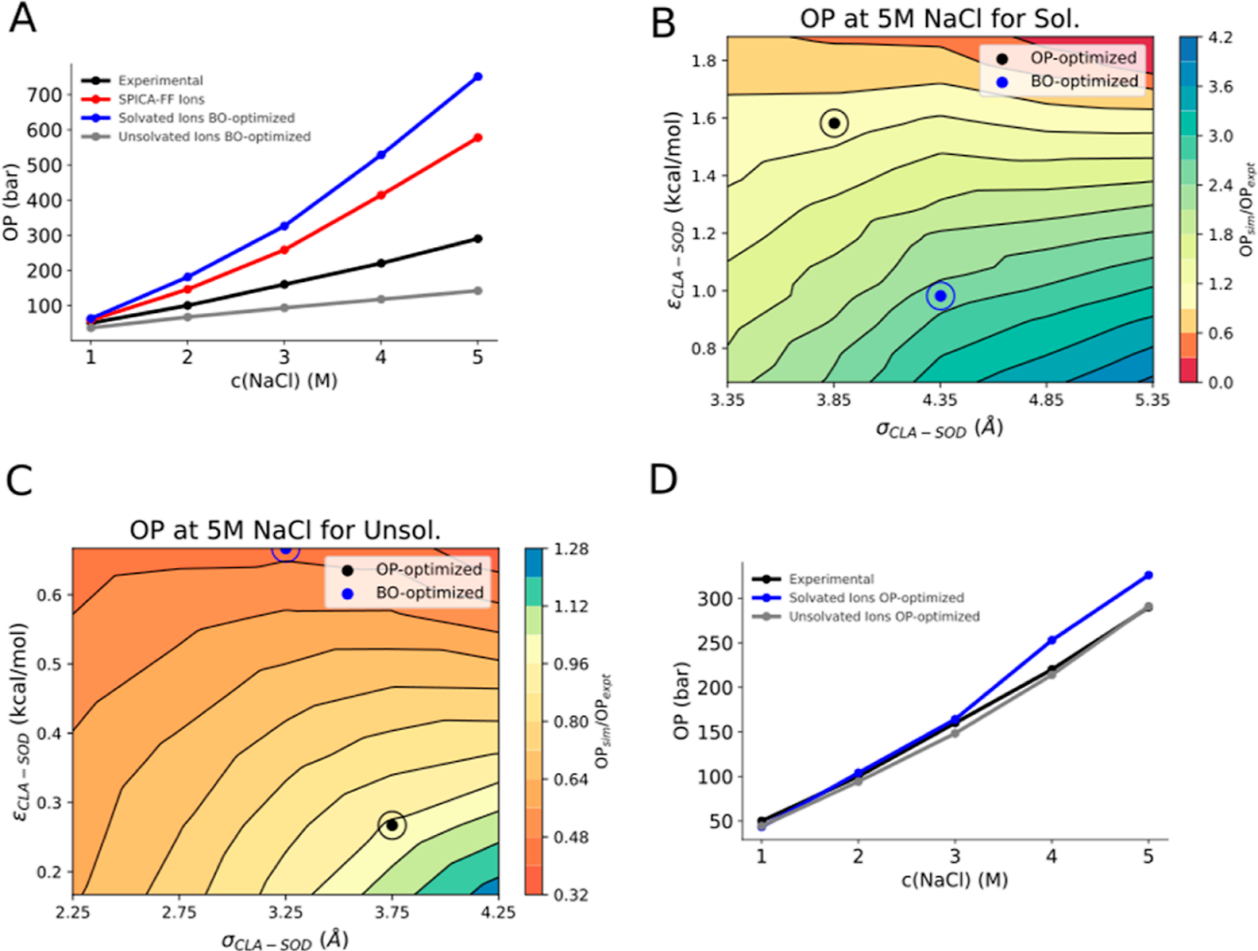
(A) OP results of the various ionic models before OP optimization and experimental measurements^30,31^ against the sodium chloride concentration. (B) Variation of the nonbonded LJ parameters of the solvated sodium and chloride cross-interactions. (C) Variation of the nonbonded LJ parameters of the unsolvated sodium and chloride cross-interactions. For both (B) and (C), blue dots represent the initially BO-optimized parameter values which satisfies condition 1. Black dots represent the values obtained with the optimal parameter set (OP-optimized parameters) satisfying condition 2. (D) OP results of the OP-optimized ionic models and experimental measurements against the sodium chloride concentration.

To explore the impact of the ion models on the osmotic pressure, we analyzed aqueous solutions with NaCl concentrations from 1 to 5 M. Figure 3A demonstrates that none of the ion models successfully replicates the experimentally observed osmotic pressure values as the concentration varies.^30,31^ The solvated ion models, including the original SPICA-FF ions and the newly optimized solvated ions, tend to overestimate osmotic pressure, with the disparity growing as sodium chloride concentration increases. In contrast, the unsolvated ion models consistently underestimate the osmotic pressure with increasing molar concentration.

To test whether slight parameter adjustments could improve osmotic pressure reproduction, we implemented a strategy similar to that used in a previous study.^20^ This approach involved tuning the nonbonded LJ cross-interaction parameters for sodium and chloride ions in the optimized models to match the osmotic pressure values at 5 M concentration, i.e., . The parameters before (blue dots; BO-optimized parameters) and after (black dots; from now on called “osmotic pressure-optimized” or simply “OP-optimized”) optimization are shown in Figure 3B,C. Our procedure reveals that, to correctly reproduce osmotic pressure for solvated ions, ε_CLA-SOD_ needs to be increased, thus making the interaction between the sodium and chloride bead more attractive (Figure 3B). On the other hand, analysis of unsolvated ions showed that the initially optimized parameters (blue dot) yielded highly attractive LJ sodium bead and chloride bead interactions, resulting in an underestimation of the osmotic pressure. Figure 3C shows that by adjusting these optimized parameters by specifically increasing σ_CLA-SOD_ and decreasing ε_CLA-SOD_ (black dot), we could achieve accurate osmotic pressure reproduction (Figure 3D).

To ensure the robustness of the optimized parameters in accurately representing osmotic pressure, we assessed their performance at various NaCl concentrations below 5 M in water. Figure 3D demonstrates that the OP-optimized models closely match experimental data in both cases.

### 3.3 Reassessment of the Macroscopic Properties

**Figure 4:**
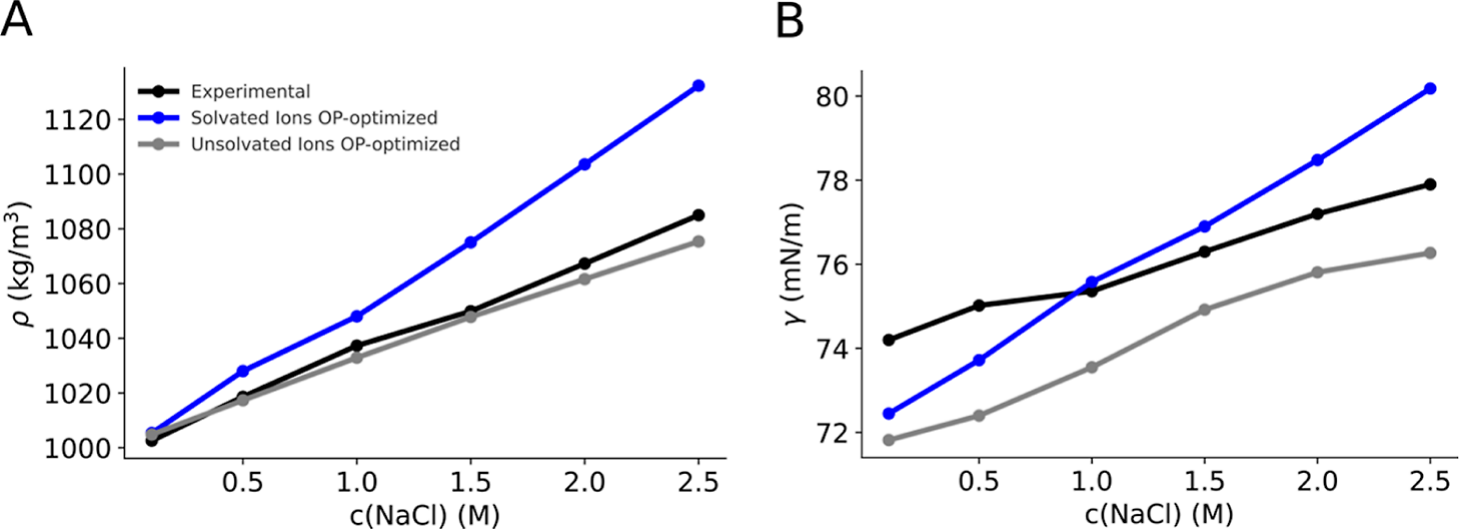
(A) Experimental and simulated density at various concentrations for the OP-optimized ionic models. Simulation error ∼1 kg/m^3^. (B) Experimental and simulated surface tension at various concentrations for the OP-optimized ionic models. Simulation error ∼0.7 to 1 mN/m.

To further assess the reliability of these OP-optimized parameters, we examined their impact on the density and surface tension reproduction. Figure 4A reveals a significant disparity in simulated density values with respect to the experimentally measured ones for solvated ions at concentrations exceeding 1.5 M. This possibly stems from the heightened attraction between sodium and chloride beads. The discrepancy in density values could be attributed to water molecules in solvated beads, which diminish the count of pure water beads with rising ionic concentration. Consequently, this reduction in screening from pure water allows for greater attraction, and substantially overestimated simulated density values are obtained at higher concentrations. Unsolvated ions do not encounter such a screening issue; hence, they show an accurate density reproduction with increase in sodium chloride concentration.

In Figure 4B, the solvated model exhibits a steep increase in surface tension with increasing concentration, albeit falling within a 7% error margin relative to experimental measurements. Notably, the gradient of the simulated surface tension curve is slightly greater than that of the experimental one. Conversely, unsolvated ions provide well-reproduced values within a 7% error and a gradient similar to that of the experimental measured curve.

Emphasizing the notion that the SPICA-FF framework is designed to capture the liquid-phase system’s density and surface tension, we represent the BO-optimized (blue dot in Figure 3B) solvated ion model as SPICA-M ions (i.e., only fulfilling the primary condition) for subsequent validation studies. However, in the case of unsolvated ions, the condition 1- and 2-optimized model (OP-optimized, i.e., satisfying both the primary and the secondary condition; black dot in Figure 3C) effectively reproduces density and surface tension. Therefore, this parameter set is employed for further investigations and is denoted as SPICA-US ions. The final parameters of the ionic models employed are provided in Tables S1–S4, in the Supporting Information.

### 3.4 Interfacial Tension Analysis

To recognize the influence of ion models on the hydrophobic effect, we computed the interfacial tension between sodium chloride solutions at various concentrations and hydrophobic molecules, such as alkanes. To do so, we conducted simulations involving decane–water and triolein–water binary mixtures, employing the original and newly optimized (SPICA-M and SPICA-US) ion models to examine their effect on the interfacial tension.

**Figure 5:**
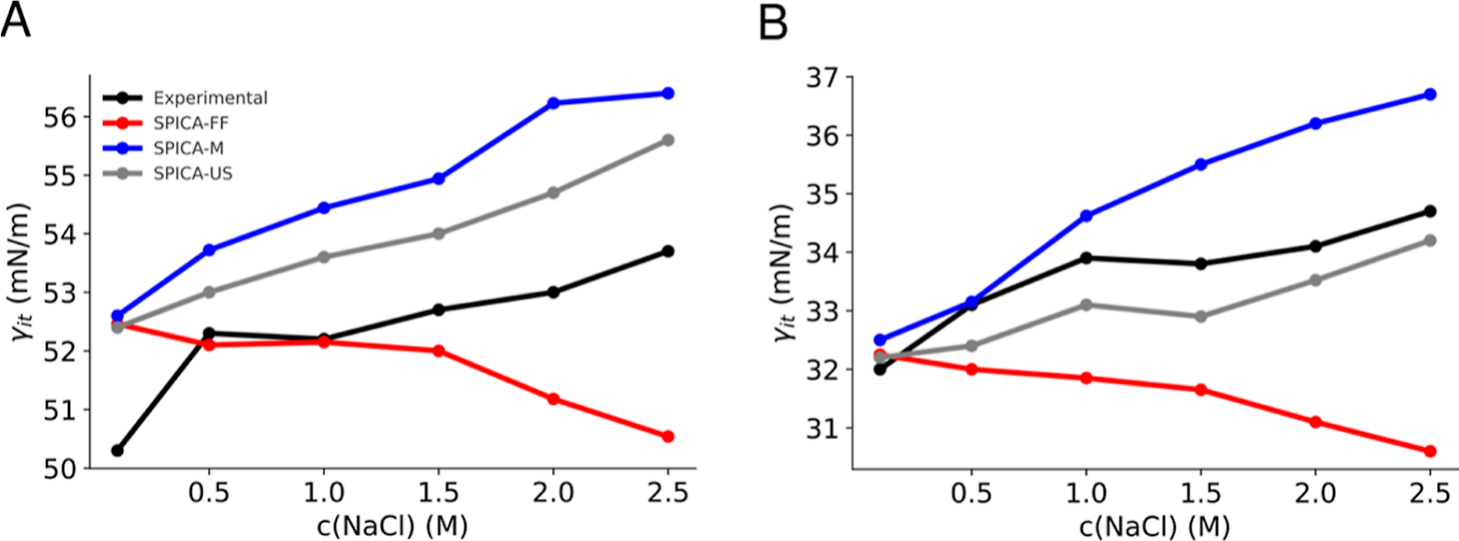
(A) Decane–sodium chloride solution liquid/liquid interfacial tension. (B) Triolein–sodium chloride solution liquid/liquid interfacial tension. Simulation error ∼0.7 to 1 mN/m. Experimental data in Table S5.

It was expected that the presence of sodium chloride would elevate interfacial tension, and such a hypothesis is consistent with experimental observations.^34,35,51^ When the sodium chloride concentration in the water phase increases, the interfacial tension in the oil phase also increases. This suggests a strengthening of the hydrophobic effect. However, as shown in Figure 5A, the original SPICA-FF model exhibits a contrasting behavior in the case of the decane–water system, indicating a decrease in the hydrophobic effect. In contrast, the SPICA-M and SPICA-US models align with the expected trend: the hydrophobic effect intensifies with higher sodium chloride concentrations, in agreement with the experimental observations.

To further confirm these results, the triolein–water interface was analyzed, as illustrated in Figure 5B. The triolein/water interface is slightly more complex due to a weakly polar ester moiety in the hydrophobic triolein. Similar to the results on the decane–water interface, the original SPICA-FF ion model pointed toward a reduced interfacial tension as the sodium chloride concentration increased. In contrast, the newly optimized ion models predicted the right trend. However, the SPICA-US model obtains a gradient equivalent to the experimental trend. Both the results reveal an intensified hydrophobic effect in line with elevated sodium chloride concentrations, also validating *a posteriori* the scaling methodology employed in this work for ion–alkane bead interactions in the case of SPICA-US ions.

### 3.5 POPC Bilayer Membrane in the Presence of Ions

**Figure 6:**
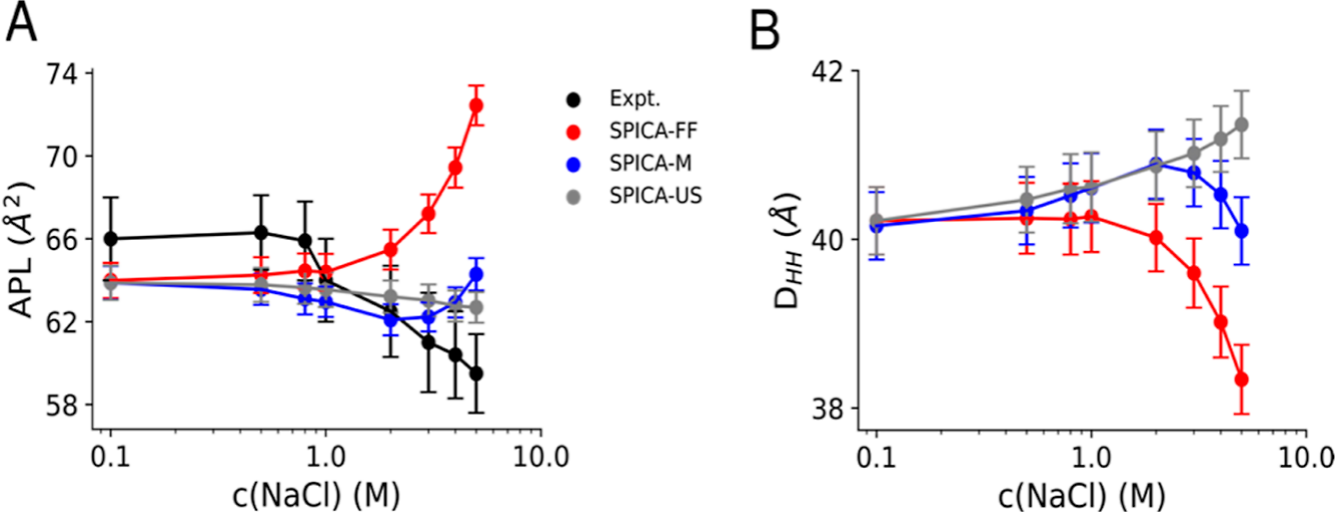
(A) APL assessment of the optimized ionic models: the original SPICA-FF, SPICA-M, and SPICA-US. (B) Bilayer thickness assessment of the SPICA-FF, SPICA-M, and the SPICA-US ion models. Experimental data taken from ref (33).

Multiple experimental studies have shown that an increase in monovalent ion concentration leads to the condensation of phospholipid bilayers, resulting in alterations of global properties like the APL and bilayer thickness.^33,52,53^ To further validate the applicability of the newly optimized ion models and compare them with the original SPICA-FF, we investigated the effects of sodium and chloride ionic solutions of varying concentrations on zwitterionic POPC bilayers. In Figure 6A, we present our findings regarding APL in response to increasing sodium chloride concentration. The experimental data demonstrates an apparent reduction in APL with increasing salt concentration, indicating bilayer compactification.^33^

Intriguingly, the three CG models (original SPICA-FF, SPICA-M, and SPICA-US) agree well with experimental APL values up to 1 M sodium chloride concentration. However, disparities emerge at concentrations beyond 1 M. Contrary to experimental trends, the original SPICA-FF model notably diverges, revealing an expansion of the bilayer as salt concentration increases. On the other hand, SPICA-M initially demonstrates a decrease in APL from 1 to 2 M concentration but exhibits an increase for concentrations exceeding 2 M. This shift is attributed to the larger bead size of the solvated ions, potentially leading to crowding at the interface and an increase in lipid spacing. Meanwhile, the SPICA-US model consistently records a decrease in APL with increasing sodium chloride concentration owing to the smaller bead size of the unsolvated ions. Nevertheless, the gradient of this simulated curve is slightly lower than that of the experimental curve, resulting in qualitative agreement.

Moreover, the experimental work^33^ has indicated that a reduction in APL corresponds to bilayer swelling involving transverse expansion accompanied by lateral compression. To explore this phenomenon further, we calculated the bilayer thickness as the lipid’s transverse^31^ headgroup-to-headgroup distance (*D*_HH_). Figure 6B reveals that the original SPICA-FF model decreases the bilayer thickness, aligning with membrane expansion, contrary to experimental findings. In the case of the SPICA-M ion model, the bilayer thickness increases up to a 2 M concentration, corresponding to the APL decrease. However, at higher concentrations, the increase in the APL leads to a decrease in bilayer thickness. Conversely, the SPICA-US model consistently exhibits an increase in bilayer thickness with rising sodium chloride concentration. Although quantitative comparison with experiments is challenging due to variations in data presentation, qualitative alignment with experimental trends is evident.

**Figure 7:**
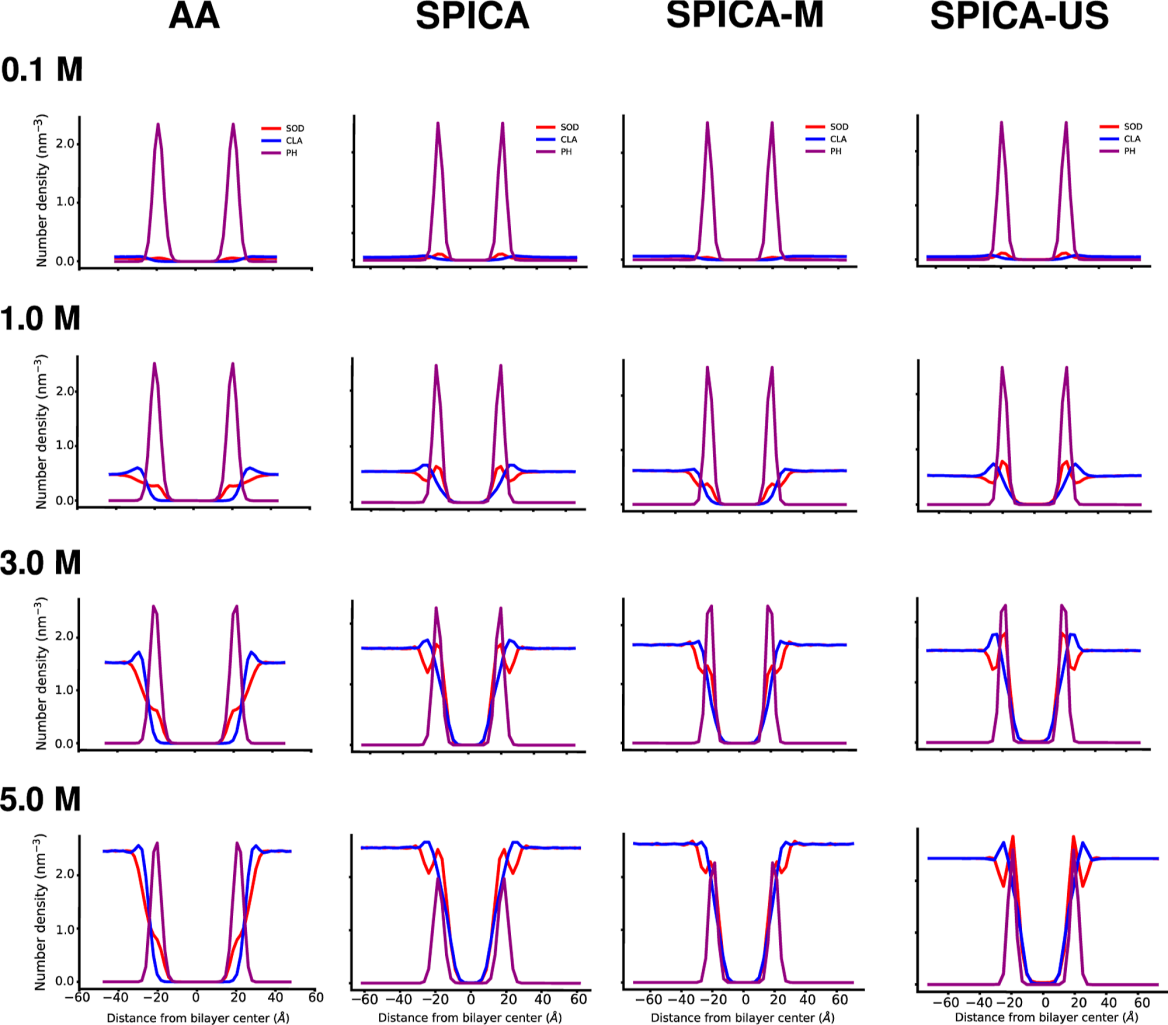
Density distributions of phosphate (PH), sodium (SOD), and chloride (CLA) beads in simulations of the POPC bilayer in the presence of sodium chloride ions at varying concentrations. Atomistic (AA) simulations employed the CHARMM36 FF, while CG simulations utilized the SPICA FF with the various ion models.

Furthermore, analysis of the density distributions of ions in the presence of bilayers (Figure 7) shows that all models reproduce well the distribution of ions around the bilayer interface. Specifically, the original SPICA-FF and SPICA-US slightly overestimate the concentration of ions in the proximity of the lipid phosphate layer, while the SPICA-M model shows remarkably good agreement with the atomistic results (Figure 7).

**Figure 8:**
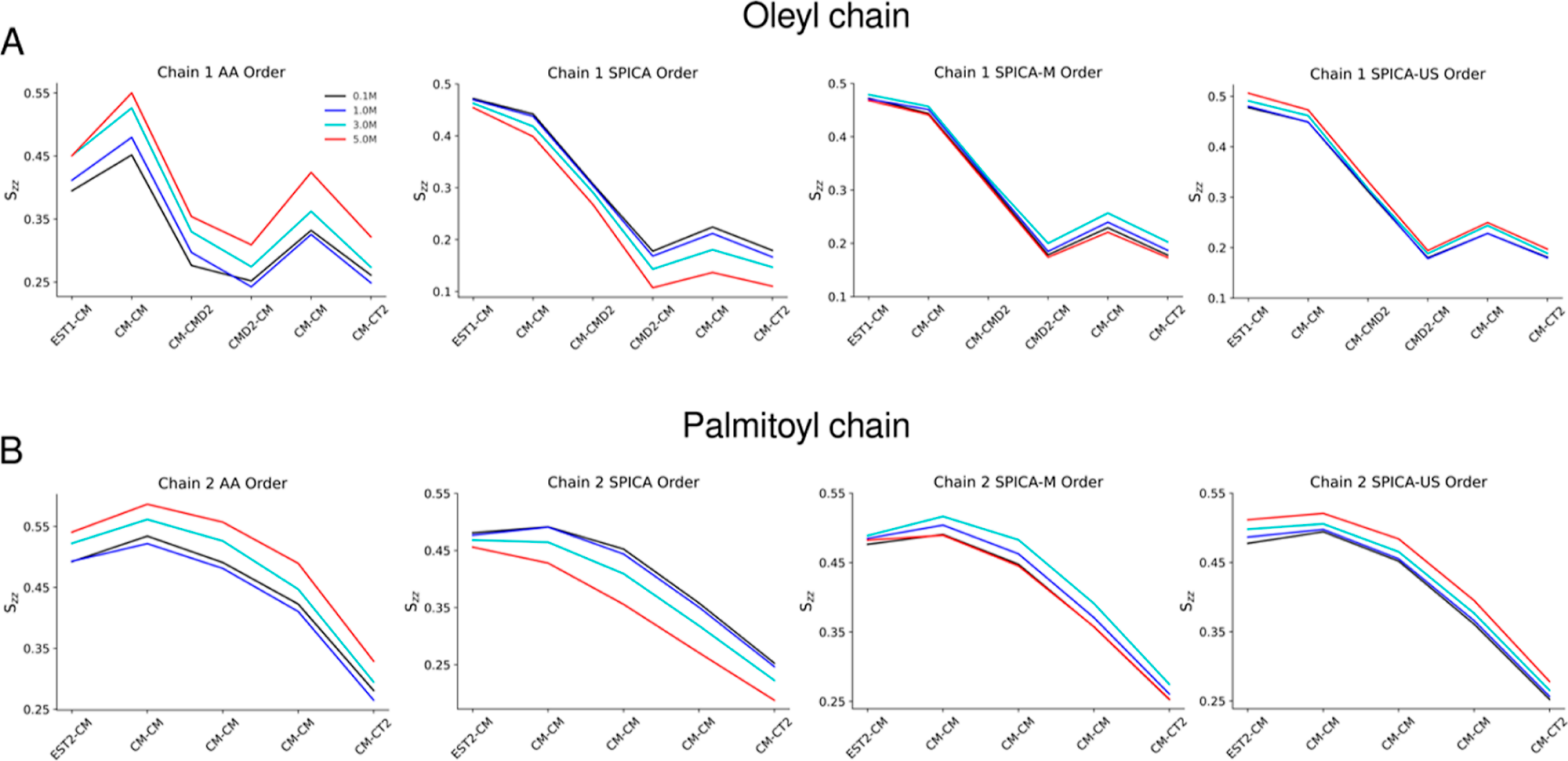
Order parameters for POPC oleyl (A) and palmitoyl chain (B) for the following models: the CG-mapped CHARMM 36 FF, the original SPICA-FF, SPICA-M, and SPICA-US, at various ionic concentrations.

Next, we computed the order parameters for the POPC lipid acyl chains. In AA simulations, carried out with the CHARMM 36 FF, rigidification of the POPC bilayer with the increase in salt concentration is observed.^54^ The lipid chain ordering for the CG-mapped AA trajectories shows an upward trend with the increase in salt concentrations for both the oleyl and palmitoyl chain (Figure 8A,B). However, at the CG level, significant differences in the three profiles can be observed, as shown in Figure 8A,B. For the original SPICA-FF ion model, the ordering progressively decreases as a function of ion concentration for both the oleyl and palmitoyl chains of POPC. This indicates that in the original ion model, the acyl chains are more disordered with the increase in concentration due to the progressive expansion of the bilayer. In the case of the SPICA-M model, the increase in ion concentration up to 3.0 M corresponds to an increase in acyl chain ordering for both chains. However, the inversion of the trend is reflected at very high concentrations due to the expansion of the bilayer, mostly arising due to steric clashes. Finally, for the SPICA-US ion model, there is a clear upward trend with the increase in concentration for both acyl chains, implying the rigidification of the bilayer.

Lastly, as a further test, we analyzed the behavior of the three ion models in the presence of anionic lipids, specifically POPS. We implemented two bilayer compositions: 4:1 POPC:POPS and 3:2 POPC:POPS, in the presence of 1 M sodium chloride. Tables S7 and S8 show that although all three models exhibit a decrease in APL compared to a pure POPC bilayer in the presence of sodium chloride, the SPICA-M and SPICA-US models align more closely with AA results. Similarly, as in the case of POPC, the SPICA-M model also shows improved agreement with the AA simulations for what pertains the distribution of ions close to the lipid bilayer (Figure S1).

## 4 Conclusions

In this work, we developed two optimized models for ions consistent with the SPICA CG-FF, namely, SPICA-M and SPICA-US. These models show improved accuracy, particularly in replicating physicochemical properties of sodium chloride solutions, such as density and surface tension. Unlike the original parameters, these models accurately capture the increasing hydrophobic interactions in the presence of sodium chloride, as highlighted by their ability to reproduce the correct interfacial tension between nonpolar molecules, such as alkanes and ionic solutions. In more complex systems such as phospholipid bilayers, these optimized models lead to changes in the properties of phospholipid bilayers in the presence of sodium chloride, which is in agreement with experimental data. Overall, these refinements bring the model closer to the experimental data, enhancing its reliability.

Our results highlight the ability of the newly optimized solvated ion model (SPICA-M) to qualitatively capture the behavior of lipid bilayers in the presence of sodium chloride concentrations smaller than 2 M, improving upon the original SPICA-FF parameters. At concentrations exceeding 2 M, the SPICA-US model displays promising agreement with experimental trends. Notably, the SPICA-US model also shows high accuracy when simulating osmotic pressure.

SPICA-M and SPICA-US represent a significant step forward in understanding ion behavior, yet they exhibit certain limitations. Further research and validation efforts are necessary to unlock their full potential, for example, in the field of intrinsically disordered proteins (IDPs). It is known that IDPs interact diversely with various ionic species at low and high concentrations, influencing the stability of condensates.^55,56^ Given the prevalent use of CG simulations to explore such large systems, we foresee that an accurate ionic model, particularly at high concentrations, will be useful in this research field. This study establishes a protocol for achieving it.

Moreover, as Go-like models are incorporated to simulate proteins and RNA in CG simulations, a promising avenue emerges for analyzing folding and unfolding dynamics at the CG level. Recognizing the role of ion charge density in protein and RNA folding by developing reliable CG ion models becomes apparent.^57^ This work represents a stride in refining ion models in CG systems, offering a pathway to delve into molecular-level interactions involving ions and potentially yielding valuable insights across various biological disciplines.

## Supporting Information Available

Bead information on the solvated and unsolvated models; parameters of the original SPICA, SPICA-M, and SPICA-US ion models; experimental surface tension, interfacial tension (decane and triolein), and density data; and APL data of POPC:POPS bilayers and density distribution of ions with the phosphates of the POPC:POPS bilayers (PDF)
