# Supplementary material for: A Coarse-Grained SPICA Makeover for Solvated and Bare Sodium and Chloride Ions
Source: J Chem Theory Comput. 2024 Aug 19;20(17):7624–34. doi: 10.1021/acs.jctc.4c00529 (PMC11391577; doi:10.1021/acs.jctc.4c00529)
Supplement: Supplementary file 1 — ct4c00529_si_001.pdf [file ct4c00529_si_001.pdf]

Supporting information

A coarse-grained SPICA makeover for solvated and bare sodium and chloride ions

Janak Prabhu[1], Matteo Frigerio[2], Emanuele Petretto[1], Pablo Campomanes[1], Stefan Salentinig[2,3] and Stefano Vanni[1,3]*

[1]Department of Biology, University of Fribourg, 1700, Fribourg, Switzerland

[2]Department of Chemistry, University of Fribourg, 1700 Fribourg, Switzerland

[3]National Center of Competence in Research Bio-inspired Materials, University of Fribourg, 1700 Fribourg, Switzerland

*Email – stefano.vanni@unifr.ch

Supplementary Information

Bead Types

Molecule	Type	Solvated mass	Unsolvated mass
Water	WAT	54.0460	54.0460
Sodium ion	SOD	77.0342	22.9900
Chloride ion	CLA	71.4796	35.4530

Table S1. Bead types and mass for water, sodium and chloride ion CG beads.

1. Original SPICA-FF parameters

Bead 1	Bead 2	ε (kcal/mol)	σ (Å)
WAT	WAT	0.895	4.371
WAT	SOD	0.895	4.371
WAT	CLA	0.895	4.371
SOD	CLA	0.895	4.371
SOD	SOD	0.350	4.371
CLA	CLA	0.350	4.371

Table S2. Parameters of the original SPICA-FF sodium chloride ions with the water interactions.

2. SPICA-M parameters

Bead 1	Bead 2	ε (kcal/mol)	σ (Å)
WAT	WAT	0.895	4.371

WAT	SOD	1.003	4.491
WAT	CLA	1.007	4.410
SOD	CLA	0.982	4.352
SOD	SOD	0.395	4.325
CLA	CLA	0.362	4.355

Table S3. Parameters of the SPICA-M sodium chloride ions with the water interactions.

3. SPICA-US parameters

$\kappa_{SOD}(\epsilon) = 0.6235$; $\kappa_{SOD}(\sigma) = 0.7378$ $\kappa_{CLA}(\epsilon) = 0.8011$; $\kappa_{CLA}(\sigma) = 0.8598$

**Bead 1**	**Bead 2**	**ε (kcal/mol)**	**σ (Å)**
WAT	WAT	0.895	4.371
WAT	SOD	0.558	3.225
WAT	CLA	0.717	3.758
SOD	CLA	0.2671	3.7500
SOD	SOD	0.498	2.876
CLA	CLA	0.2975	4.1405

Table S4. Parameters of the SPICA-US sodium chloride ions with the water interactions.

4. Experimental surface tension

**Substrate**	**Surrounding Medium**	**Conc. (mol/L)**	**Surface tension (mN/m)**
Water	Air	-	72.8 ± 0.1
NaCl	Air	0.1	74.2 ± 0.1

NaCl	Air	0.5	75.05 ± 0.02
NaCl	Air	1.0	75.36 ± 0.04
NaCl	Air	1.5	76.3 ± 0.1
NaCl	Air	2.0	77.2 ± 0.1
NaCl	Air	2.5	77.9 ± 0.1
NaCl	Air	3.0	79.5 ± 0.1
Decane	Water	-	50.3 ± 0.4
Decane	NaCl	0.5	52.3 ± 0.2
Decane	NaCl	1.0	52.2 ± 0.2
Decane	NaCl	1.5	52.7 ± 0.1
Decane	NaCl	2.0	53.0 ± 0.1
Decane	NaCl	2.5	53.7 ± 0.2
Triolein	Water	-	32.0 ± 0.1
Triolein	NaCl	0.5	33.1 ± 0.2
Triolein	NaCl	1.0	33.9 ± 0.3
Triolein	NaCl	1.5	33.8 ± 0.2
Triolein	NaCl	2.0	34.1 ± 0.1
Triolein	NaCl	2.5	34.7 ± 0.2

Table S5. Experimental values for the surface tension of NaCl aqueous solutions and interfacial tension for decane (triolein) / NaCl.

5. Experimental density

Substrate	Density (g/mL)	Concentration (mol/L)
Water	0.9982	-
Air	0.0012929[1]	-

NaCl	1.0026	0.1
NaCl	1.0186	0.5
NaCl	1.0373	1.0
NaCl	1.0499	1.5
NaCl	1.0673	2.0
NaCl	1.0850	2.5
NaCl	1.1140	3.0
Decane	0.7301	-
Triolein	0.9182	-

Table S6. Experimental values for the density.

**Model**	**APL (Å^2)**
AA	59.84 ± 0.43
SPICA	62.61 ± 0.81
SPICA-M	61.20 ± 0.72
SPICA-US	61.10 ± 0.74

Table S7. APL of POPC:POPS (4:1) in the presence of 1M sodium chloride.

**Model**	**APL (Å^2)**
AA	56.92 ± 0.38
SPICA	61.17 ± 0.72
SPICA-M	60.33 ± 0.71
SPICA-US	59.28 ± 0.76

Table S8. APL of POPC:POPS (3:2) in the presence of 1M sodium chloride.

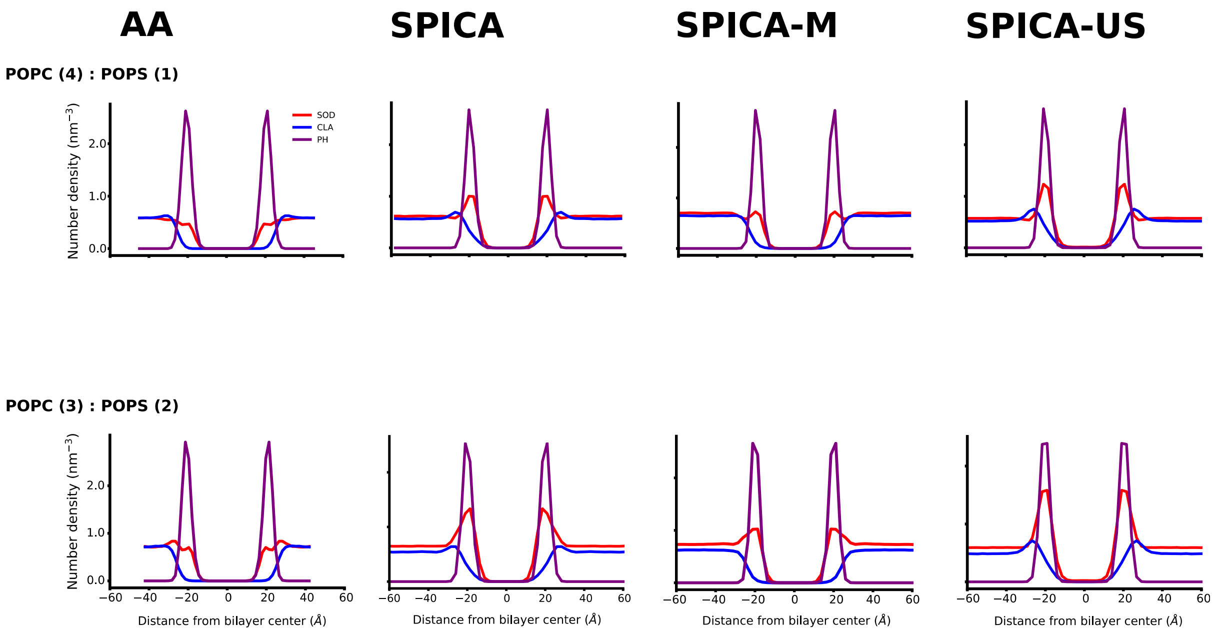

Figure S1. Density distributions of phosphate (PH), sodium (SOD) and chloride (CLA) beads for POPC:POPS bilayers in the presence of sodium chloride ions at 1M concentration. Atomistic (AA) simulations employed the CHARMM36 force field, while coarse-grained (CG) simulations utilized the SPICA force field with the various ion models.

References

1. Davis, Joseph R. Metals handbook desk edition (1998)
